# The Diagnostic Challenge of Seronegative Autoimmune Encephalitis With Super-Refractory Status Epilepticus

**DOI:** 10.7759/cureus.11587

**Published:** 2020-11-20

**Authors:** Koti Vadagandla, Vinay Jahagirdar, Kaanthi Rama

**Affiliations:** 1 Anesthesia, Medicover Hospital, Hyderabad, IND; 2 Medicine, Gandhi Medical College and Hospital, Secunderabad, IND

**Keywords:** autoimmune, encephalitis, status epilepticus, seronegative encephalitis, epileptic seizures, refractory seizure

## Abstract

Autoimmune encephalitis is an immune-mediated syndrome, with sub-acute to chronic presentations, such as memory impairment, altered sensorium, behavioral abnormality, psychosis, and seizures. It poses a two-fold diagnostic challenge: firstly, because of its variable clinical presentation and secondly, due to the wide variety of autoimmune antibodies causing it, which makes it difficult to identify the underlying etiology. Treatment should not be delayed due to pending laboratory workup, as early recognition and initiation of therapy prevents long term neurological sequelae. This is a case report of a 59-year-old female who presented with neuropsychiatric symptoms, which evolved into refractory status epilepticus and autonomic dysfunction, requiring anesthesia induced coma. While her MRI had positive findings of encephalitis, she tested negative for infectious diseases and antibody panels.

## Introduction

Autoimmune encephalitis syndromes have a wide clinical spectrum ranging from typical limbic encephalitis to syndromes with complex neuropsychiatric symptoms, such as the loss of memory, cognition, psychosis, seizures, abnormal movements, or coma. Its etiology can be broadly divided into three groups. In the first group, antibodies are produced against tumor antigens in a paraneoplastic syndrome. In the second group, antibodies are produced against extracellular and intracellular ion channels and proteins. In the third group, antigens are not clearly established. 

Imaging abnormalities include medial temporal lobe changes and contrast-enhancing abnormalities in cortical or subcortical regions. Electroencephalography (EEG) shows infrequent epileptic activity but a frequent, slow, disorganized activity that does not correlate with most abnormal movements. A unique EEG pattern called extreme delta brush is seen in prolonged illness [1].

Seronegative autoimmune encephalitis is a subcategory of autoimmune encephalitis diagnosed when autoimmune antibodies are not detected in cerebrospinal fluid (CSF) or serum [2-3]. The possible reasons for the absence of antibodies, as stated by Najjar et al., include declining serum antibodies and the existence of unidentified antibodies which are yet to be discovered [2]. Early recognition and treatment prevent relapse and reduce long-term neurological sequelae, as proposed by Darnell and Posner [4]. Management of autoimmune encephalitis is mainly by using immunosuppressants. First-line therapy includes steroids and intravenous immunoglobulins (IVIG). Second-line immunotherapy, such as rituximab or cyclophosphamide, should be considered if the symptoms do not subside with the first-line therapy. 

## Case presentation

A 59-year-old female patient with no significant past medical history presented to the emergency room with a history of fever, headache, drowsiness, and body pains for one week. Suspecting viral encephalitis, acyclovir was initiated. The patient had one episode of generalized tonic-clonic seizures (GTCS) post-admission. Magnetic resonance imaging (MRI) of the brain ruled out a cerebrovascular accident. CSF analysis did not show features of meningoencephalitis. Serum sodium was 122 meq/mol. Hyponatremia was suspected as the cause of the GTCS, and the patient was started on 3% normal saline. The patient recovered, and a Glasgow Coma Scale score of 15 was noted. However, six hours later, she became drowsy and went into respiratory failure with a partial pressure of carbon dioxide (PaCO_2_) of 55, prompting non-invasive ventilatory support. The patient had one more episode of GTCS. On examination following that episode, she had altered sensorium, disorientation, confusion, and faciobrachial dystonic seizures (FBDS), hinting at autoimmune encephalitis. A serum autoimmune panel was ordered, which came back negative. Three anti-epileptic drugs, namely, sodium valproate, phenytoin, and levetiracetam, were started. However, the patient continued to have recurrent complex partial seizures. She developed hyperthermia, tachycardia, and hypertension, indicating autonomic dysfunction. Infectious causes, including herpes simplex, chikungunya, cytomegalovirus, and dengue were ruled out. Procalcitonin was normal. The autonomic dysfunction was treated with nitroglycerine and dexmedetomidine infusion. Despite this, the patient had continuous faciobrachial dystonia and cataplexy. Methylprednisolone, 1 g/day, was started. Paraneoplastic causes were ruled out through CT scans and tumor markers. The patient required ventilator support because of recurrent status epilepticus and autonomic instability. As she continued to have recurrent seizures despite steroids, intravenous immunoglobulins were initiated. The patient had persistent seizures on the ventilator with four antiepileptic drugs (levetiracetam, phenytoin, sodium valproate, clonazepam), and propofol infusion of 1.5 mg/kg/hr. A diagnosis of super-refractory status epilepticus was made because of persistent seizures after 24 hrs of propofol infusion. Repeat MRI of the brain showed medial temporal lobe hyperintensity. Thiopentone, 3 mg/kg/hr, was started for burst suppression and the dosage was increased to a maximum of 6 mg/kg/hr. Midazolam, 2 mg/hr, was continued, along with thiopentone, and an intermittent bolus of thiopentone was given.

After three days of thiopentone infusion, the EEG showed a generalized, reduced spike pattern. The thiopentone dose was reduced and eventually stopped, but she continued to have faciobrachial dystonia. Suspecting N-methyl-D-aspartate (NMDA)-receptor encephalitis, magnesium sulfate, 1 g/hr, and ketamine infusion, 1.5 mg/kg/hr, were started and were continued for one week. The FBDS persisted, prompting the initiation of second-line immunotherapy with rituximab, 625 mg (at 375 mg/m^2^) with serial CD19 and CD20 antibody monitoring. 

The patient improved neurologically with reduced spikes on EEG. Eventually, two weeks down the line, the patient was discharged without any neurological or cognitive abnormality. 

The investigations and timeline of events during the hospital stay are depicted in Tables 1-2, respectively.

**Table 1:** Chronology of Laboratory and Imaging Investigations Ordered AMPA: α-amino-3-hydroxy-5-methyl-4-isoxazolepropionic acid: CMV: cytomegalovirus; CSF: cerebrospinal fluid; EEG: electroencephalography; GABA: gamma-aminobutyric acid; HSV1: herpes simplex virus 1; HSV2: herpes simplex virus 2; MRI: magnetic resonance imaging; NMDA: N-methyl-D-aspartate; PCR: polymerase chain reaction; VGKC: voltage-gated potassium channel

Investigation	Day Sent	Report
Complete blood count	Day 1	Normal
Liver function test	Day 1	Normal
Renal function test	Day 1	Normal
MRI brain	Day 2	Normal
CSF analysis	Day 2	Normal
EEG	Day 3	Normal
CSF PCR for HSV1 and HSV2	Day 4	Normal
Serum autoimmune panel (VGKC, NMDA, GABA, AMPA)	Day 4	Negative
CMV, Dengue, Chikungunya antibodies	Day 4	Negative
Suction tip culture	Day 9	Klebsiella pneumoniae
MRI brain	Day 10	Hyperintensity in the hippocampus and medial temporal lobe
Repeat serum autoimmune panel	Day 22	Negative
Blood culture	Day 24	Coagulase-negative Staphylococcus aureus

**Table 2:** Summary of Clinical Events CMV: cytomegalovirus; CSF: cerebrospinal fluid; EEG: electroencephalography; GCS: Glasgow Coma Scale; GTCS: generalized tonic-clonic seizure; IV: intravenous; IVIG: intravenous immunoglobulin G; MRI: magnetic resonance imaging; NMDA: N-methyl-D-aspartate;

Day	Event
Day 1	One episode of GTCS. Negative CSF analysis. Acyclovir started
Day 2	Negative MRI. Hyponatremia corrected. Levetiracetam started
Day 4	One episode of GTCS. Negative viral encephalitis panel, dengue serology, CMV, chikungunya antibodies sent, serum autoimmune panel sent. Clonazepam and fosphenytoin added
Day 5	Recurrent complex partial seizures and faciobrachial dystonia. Methylprednisolone (1 g IV) started autonomic instability→nitroglycerine infusion started
Day 6	Intubated because of recurrent status epilepticus in spite, of multiple antiepileptic drugs and autonomic instability. Paraneoplastic panel sent. IVIG initiated and propofol infusion started (1.5 mg/kg/hr)
Day 8	Persistent seizures after 24 hrs of propofol infusion→diagnosed as super-refractory status epilepticus. Thiopentone (3 mg/kg/hr) and midazolam (2 mg/hr) started
Day 9	Thiopentone dosage increased (6 mg/kg/hr), according to burst suppression on EEG, along with midazolam (3 mg/hr). Autoimmune panel and viral panel negative. Suction tip culture showed Klebsiella; antibiotics started according to sensitivity
Day 10	Repeat MRI shows medial temporal lobe hyperintensity
Day 11	Seizure episodes reduced. Thiopentone dosage reduced according to EEG burst suppression and stopped
Day 13	Paramphenol started. Magnesium (1 g/hr infusion) and ketamine (1 mg/kg/hr) infusion started suspecting NMDA encephalitis
Day 18	Ketamine dose reduced and tracheostomy done
Day 22	Repeat autoimmune panel negative; patient’s neurological status improved
Day 24	Ketamine stopped. Blood culture shows coagulase-negative Staphylococcus aureus
Day 25	Initiated weaning from the ventilator
Day 30	Rituximab, first dose - 625 mg, given for faciobrachial dystonia
Day 32	EEG normal, GCS: E1VtM6
Day 36	Tracheostomy, decannulated, and speech therapy started
Day 45	Tracheostomy closed
Day 48	Patient discharged with oral antiepileptic drugs and subsequent rituximab given once a week, for the next two weeks.

## Discussion

The term seronegative autoimmune encephalitis has been used to describe a subgroup of autoimmune encephalitis cases with negative serologic tests for autoantibodies [2-3]. 

Pathophysiology of the disease can be explained by the presence of a diverse group of autoantibodies to intracellular or extracellular receptors and paraneoplastic tumor antigens, such as anti-Hu antibodies and anti-NMDA receptor antibodies [5]. There are many such unidentified receptors as reported by previous case reports [2-3]. Paraneoplastic syndromes should be considered as etiology if the patient is diagnosed to have certain tumors, evidenced by imaging and tumor markers. Some tumors associated with paraneoplastic encephalitis include immature teratoma in females and thymoma [5]. The tumor antigens act as receptors for antibodies in such cases [6-7].

Clinical presentation in autoimmune encephalitis varies based on the causative antibody [5]. Psychiatric manifestations and abnormal movements are the early presenting features in most patients. There are instances where a patient was referred to a psychiatrist in view of predominant psychiatric manifestations, leading to a delay in diagnosis, as well as early initiation of treatment which may result in persistent disabilities. 

Patients with positive antibodies to gamma-aminobutyric acid (GABA) and NMDA present with seizures and refractory status epilepticus, as mentioned by Lancaster et al. and Petit-Pedrol et al. [8-9]. FBDS are classically associated with leucine-rich glioma-inactivated 1 (LGI1) antibody [10]. Our case presented with psychiatric manifestations and behavioral abnormalities. She tested negative for GABA antibodies but developed super-refractory status epilepticus and FBDS later. Status epilepticus was earlier defined as lasting for 30 minutes or longer but is now more often defined as lasting five minutes or longer [11]. Refractory status epilepticus is defined as persistent seizures, despite the use of two intravenous medications, one of which is a benzodiazepine [12]. Super-refractory status epilepticus is defined as status epilepticus that continues or recurs 24 hours or more after the onset of anesthetic therapy. Despite aggressive treatment with four anti-epileptics and an anesthesia-induced coma, the seizures lasted for more than 48 hours in our case.

Existing criteria for autoimmune encephalitis depend on antibody testing, which may lead to a delay in diagnosis. To overcome this, Graus et al. have suggested the criteria presented in Table 3, for autoantibody-negative but probable autoimmune encephalitis [13].

**Table 3:** Criteria for Autoantibody-negative but Probably Autoimmune Encephalitis * autoimmune encephalitis can occur with normal or atypical MRI findings ** absence of pleocytosis does not rule out autoimmune encephalitis CSF: cerebrospinal fluid; IgG: immunoglobulin G Graus et al. [13]

Diagnosis can be made when all four of the following criteria have been met:	
1	Rapid progression (less than 3 months) of working memory deficits (short-term memory loss), altered mental status, or psychiatric symptoms
2	Exclusion of well-defined syndromes of autoimmune encephalitis (e.g., typical limbic encephalitis, Bickerstaff’s brainstem encephalitis, acute disseminated encephalomyelitis)
3	Absence of well-characterised autoantibodies in serum and CSF, and at least two of the following criteria: MRI abnormalities suggestive of autoimmune encephalitis,^*^CSF pleocytosis, CSF-specific oligoclonal bands or elevated CSF IgG index, or both.^**^ Brain biopsy showing inflammatory infiltrates and excluding other disorders (e.g., tumour)
4	Reasonable exclusion of alternative causes

Our patient underwent a serum antibody panel twice to check for antibodies to NMDA, GABA, and voltage-gated potassium channels (VGKC). However, the panel was negative both times. Other possible factors, including infectious, metabolic causes (like hyponatremia), and paraneoplastic tumor syndromes were ruled out. MRI of the brain showed medial temporal hyperintensity in T2 films, as documented in other similar case reports [8, 14-15]. This eventually led us to a diagnosis of seronegative autoimmune encephalitis. Constant EEG monitoring helped to monitor our case and direct treatment. EEG monitoring is necessary to exclude subclinical seizures, for prognosis, and also for management based on burst suppression [16].

Early diagnosis and intervention can improve prognosis and prevent relapses in autoimmune encephalitis [4]. So far, there is no convincing evidence to show whether IVIG or plasmapheresis is superior to the other for management of autoimmune encephalitis, and hence either may be used. If the patient remains significantly impaired after first-line therapy, second-line treatments are deployed. Second-line therapies include rituximab (often 375 mg/m^2^ weekly for four weeks) or cyclophosphamide (750 mg/m^2^ IV monthly until improvement is noted). Rituximab has a good safety profile compared to cyclophosphamide. There are no existing indications or guidelines regarding the timing of second-line immunotherapy. Our patient received Solu-Medrol® (methylprednisolone), 1 g/day for five days, and IVIG, 0.4 g/kg for five days. Due to persistent faciobrachial dystonia, rituximab was started as second-line therapy after 19 days of completion of the IVIG course. 

## Conclusions

Autoimmune encephalitis is a spectrum of disease with neurological and psychiatric symptoms. The presentation may vary based on the type of antibodies causing it. In our case, the main concerning issue was super-refractory status epilepticus, requiring continuous anesthesia to suppress EEG burst activity. This poses a diagnostic challenge as present laboratory techniques lack specificity to identify this. There are many unidentified intracellular and extracellular protein receptors; hence, a negative serology panel for antibodies should not delay the initiation of primary immunomodulatory therapy (steroids, plasmapheresis). A strong clinical suspicion of disease, after a panel to rule out paraneoplastic and infectious serology, supported by MRI imaging, is the mainstay to identify seronegative autoimmune encephalitis. Primary therapy should be initiated to prevent disease progression. If the patient continues to have symptoms despite steroids and IVIG, then second-line immunotherapy with agents like rituximab can be used. Our patient recovered well after second-line immunotherapy and was discharged without any cognitive or neurological symptoms.
